# Comparison of Long-Term Outcomes of Postmastectomy Radiotherapy between Breast Cancer Patients with and without Immediate Flap Reconstruction

**DOI:** 10.1371/journal.pone.0148318

**Published:** 2016-02-10

**Authors:** Hsin-Hua Lee, Ming-Feng Hou, Shu-Yi Wei, Sin-Daw Lin, Kuei-Hau Luo, Ming-Yii Huang, Fu Ou-Yang, Chih-Jen Huang

**Affiliations:** 1 Division of Radiation Oncology, Department of Radiation Oncology, Kaohsiung Medical University Hospital, Kaohsiung Medical University, Kaohsiung, Taiwan; 2 Department of Radiation Oncology, Antai Tian-Sheng Memorial Hospital, Pingtung, Taiwan; 3 Faculty of Medicine, College of Medicine, Kaohsiung Medical University, Kaohsiung, Taiwan; 4 Department of General Surgery, Kaohsiung Medical University Hospital, Kaohsiung, Taiwan; 5 National Sun Yat-Sen University-Kaohsiung Medical University Joint Research Center, Kaohsiung, Taiwan; 6 Department of Internal Medicine, Kaohsiung Municipal United Hospital, Kaohsiung, Taiwan; 7 Yuh-Ing Junior College of Health Care & Management, Kaohsiung, Taiwan; 8 Department of Occupational and Environmental Medicine, Kaohsiung Medical University Hospital, Kaohsiung Medical University, Kaohsiung, Taiwan; Taipei Medical University, TAIWAN

## Abstract

**Purpose:**

To compare the long-term clinical outcomes of postmastectomy radiotherapy (PMRT) between breast cancer patients with and without immediate transverse rectus abdominis myocutaneous (TRAM) flap reconstruction.

**Methods:**

The study included 492 patients with stage II or III breast cancer who underwent modified radical mastectomy (MRM) and chemotherapy followed by PMRT between 1997 and 2011. Cox regression model and Kaplan-Meier curves were calculated, and the log-rank test was used to evaluate the differences between overall and disease-free survival rates in the 2 groups.

**Results:**

Among 492 patients, 213 patients had immediate TRAM flap reconstruction. The mean follow-up was 7.2 years (range, 11–191 months). The 5-year and 10-year disease free survival rates were 81% and 76% for the TRAM flap group and 78% and 73% for the non-flap group. The 5-year and 10-year overall survival rates were 89% and 73% for the TRAM flap group and 83% and 74% for the non-flap group.

**Conclusions:**

There exists no statistically significant difference in the rates of local recurrence, distant metastasis, disease-free and overall survival when comparing immediate TRAM flap reconstruction with no reconstruction. Our results suggest that immediate TRAM flap reconstruction does not compromise long term clinical outcomes in breast cancer patients requiring PMRT.

## Introduction

Surgical intervention for patients with breast cancer includes breast conserving surgery and mastectomy. The randomized trials of Danish Breast Cancer Cooperative Group, 82b and 82c, demonstrated that patients who underwent modified radical mastectomy (MRM) and received postmastectomy radiotherapy (PMRT) have a lower 10-year rate of local regional recurrence and an additional survival advantage associated with PMRT [1, 2]. Another prospective Canadian research also supported the addition of PMRT to chemotherapy after MRM since PMRT decreases rates of locoregional and systemic relapse and reduces mortality from breast cancer [3]. A meta-analysis from Early Breast Cancer Trialists’ Collaborative Group demonstrated that the survival benefit of PMRT was applicable to a subset with 1 to 3 positive lymph nodes [4–7]. From these landmark studies, current indications for PMRT at our institution include large tumor size (larger than 4 cm), positive or close surgical margins or any documented lymph node involvement.

Patients who underwent MRM have to face not only psychological trauma regarding loss of important sexual characteristics but also the deformity of body figure causing further difficulty of daily dressing. It has been the policy of our institution to perform immediate breast reconstruction on breast cancer patients who had elected reconstruction after MRM. These patients received both surgeries at one single time without the need for contralateral vertical mastopexy for symmetry [8, 9]. There are not only cosmetic but psychological advantages over conventional methods of delayed reconstruction [10–12].

However, breast reconstruction is not reimbursed by the National Health Insurance system in Taiwan. There is no mandated insurance coverage of reconstruction following mastectomy as in the United States by the Federal Breast Cancer Reconstruction Law since 1998 [13]. Patients in Taiwan who consider breast reconstruction after MRM must take not only the expense into account, but the frequent concern as to whether flap reconstruction will interfere with long-term survival or not.

Some recent studies in North American have revealed that immediate flap reconstruction is related to increased breast cancer survival yet the mechanism is still under investigation [14–16]. This might be just patient selection with in general more favorable patients undergoing immediate reconstruction. However, there has been no study focusing on evaluation of the long-term survival rates with PMRT after immediate reconstruction in Asian population showing such positive result. As health-care providers, it is our social responsibility to allow the clinicians and patients to make better clinical decisions.

To keep the patients’ best interest in mind, the objective of the current study is to identify any clinical or pathologic characteristics associated with improved survival and determine the influence of immediate autologous tissue breast reconstruction on survival in breast cancer patients receiving PMRT.

## Materials and Methods

The present study is an approved Kaohsiung Medical University Hospital Institutional Review Board clinical protocol (KMUH-IRB-990069).

### Patients

A series of 747 consecutive female patients who underwent MRM followed by post-operative adjuvant chemotherapy (CT) and then PMRT between January 1997 and December 2011 were observed retrospectively. Excluded were 255 patients with any one of the following features: a history of previous irradiation to the thorax, or age over 60 year-old, stage I or IV, or any previous cancer history, or diagnosis of synchronous contralateral breast cancer (i.e., breast cancer diagnosed in both breasts simultaneously or within a 3-month period of diagnosis of the first tumor), use of primary systemic CT or loss of follow-up after treatment due to personal reasons.

The following data were collected: age at diagnosis, information regarding primary surgery, adjuvant CT and hormonal treatment, regional PMRT method, histological diagnosis and grading, hormonal receptor status, local recurrence and distant metastases. All stages were determined according to the American Joint Committee on Cancer (AJCC) staging system, 6^th^ edition.

All patients underwent pretreatment workups, including history and physical examination, bilateral diagnostic mammograms or ultrasound, complete blood count and platelets, liver function tests, serum carcinoembryonic antigen and CA-153 tests, chest radiography, abdominal ultrasound and technetium-99 bone scintigraphy. Additionally, counseling regarding breast reconstruction was given prior to MRM. Some of the patients with T2N1 disease preferred mastectomy followed by reconstruction whereas breast conservation surgery would have been chosen.

### Ethics statement

The present study (KMUH-IRB-990069) was conducted under compliance of the Kaohsiung Medical University Hospital institutional review board regulations. All patients provided written informed consent for treatment prior to surgery and RT. Patient information was anonymized and de-identified before analysis. All data were analyzed anonymously and retrospectively.

### Surgery

All enrolled 492 patients had MRM with pathological stage II or III. They were all informed about the option of breast reconstruction. There were 213 patients (43.3%) in the transverse rectus abdominis myocutaneous (TRAM) flap group who underwent TRAM flap reconstruction immediately after MRM performed by one attending plastic surgeon (S.D.L.). The remaining 279 patients (56.7%) who chose no reconstruction were grouped as non-flap group. There was no tissue expander or implant used in TRAM-flap group.

### Systemic therapy

All patients had anthracycline with or without taxane based CT regimens. Adjuvant hormonal therapy was indicated for all hormonal receptor-positive patients. Depending on menopausal status, tamoxifen or aromatase inhibitors were given after the completion of CT.

### Radiotherapy

All patients received PMRT which was delivered at a mean dose of 50 (range 48–54) Gy in a daily fraction of 1.8–2 Gy. All dose schedules were given 5 days per week. All patients underwent Computerized Tomography simulation for three-dimensional conformal planning.

The target included the ipsilateral chest wall, mastectomy scar, the supraclavicular or infraclavicular, with or without axillary nodes and drain sites where possible. When axillary nodal dissection was not complete, axillary nodal basins was included. The radiation field encompassed a volume that included the breast reconstruction in the TRAM flap group. Computerized Tomography-based treatment planning was performed in order to identify lung and heart volumes, and minimize exposure of these organs. Treatment planning with 6MV photon used tangential beams and a series of segments produced to remove the hot spots from the open beams.

### Evaluation

End points were disease-free survival (DFS) and overall survival (OS). In general, patients were observed with standardized follow-up every 3 months after therapy for the first year, then subsequently every 6 months thereafter. The length of follow-up was defined as the time from MRM to the date of local failure or last follow-up. Local failure was defined as any disease recurrence within supraclavicular or infraclavicular region, axilla and chest wall. Any failure outside these regions was classified as a distant metastasis. DFS and OS were defined as the time interval from surgery until any type of recurrence or death from any cause, respectively. Recurrence was confirmed pathologically by surgical resection, biopsy, or cytology, and/or radiological findings, which increased in size over time. Distant metastasis was recorded mostly according to chest radiography, abdominal ultrasonography, computed tomography of the chest or technetium-99 bone scintigraphy.

### Statistical analysis

The dataset was stratified and outcomes were compared by t test or Chi-squared test. Univariate analyses were used to examine the following characteristics and its potential association with local recurrence and distant metastasis: reconstruction, age, laterality, tumor location, pathological cell pattern, pathological tumor and nodal classification, stage, estrogen receptor (ER) and progesterone receptor (PR) status, mean interval from surgery to adjuvant CT or RT. DFS and OS were assessed by Kaplan-Meier methods. A multivariate Cox proportional hazards regression was used to evaluate the association between treatment and outcome, with adjustment for clinicopathologic tumor characteristics known to be associated with relapsing disease. Estimated risks of death were calculated using hazard ratios (HR) with 95% confidence intervals (CIs).

The level of statistical significance was set at p < 0.05; all reported p values were two-tailed. The analyses were performed using the SPPS software package, version 19.0 for Windows (SPSS, Chicago, IL, USA).

## Results

Table 1 summarizes the clinicopathologic characteristics of 492 patients. There was no significant difference in terms of follow-up time, laterality, tumor location, pathological cell pattern, pathological tumor and nodal classification, stage, ER and PR status between the two groups. However, the age and the mean interval between MRM and chemotherapy (CT) were different. Younger age (44.8 vs 49.1 years, P< 0.001), longer mean interval from MRM to CT (1.25 vs. 0.76 months, P< 0.001) and that from MRM to PMRT (6.54 vs. 6.12 months, P< 0.001) were noted in the TRAM flap group.

**Table 1:** Clinicopathologic characteristics of 492 breast cancer divided by transverse rectus abdominis myocutaneous (TRAM) flap reconstruction.

	TRAM Group	Non- flap Group	*p*
**No. of cases**	213	279	
**Duration of collection**	January of 1997 to December of 2011		
**Follow-up, months (mean, median)**	11–189 (85.2,80)	12–191 (87.5,78)	0.605 ^a^
**Mean Age, years (range)**	44.80 (27–60)	49.11 (27–60)	<0.001 ^a^
**< 50**	154 (72%)	137(49%)	<0.001 ^b^
**≧ 50**	59 (28%)	142(51%)	
**Laterality**			0.716 ^b^
**Left**	110(52%)	139(50%)	
**Right**	103(48%)	140(50%)	
**Location**			0.839 ^b^
**Lateral**	154(72%)	204(73%)	
**Central/medial**	59(28%)	75(27%)	
**Pathology**			0.172 ^b^
**Infiltrating ductal carcinoma**	192(90%)	238(85%)	
**Infiltrating lobular carcinoma**	12(6%)	22(8%)	
**Medullary carcinoma**	4(2%)	3(1%)	
**Others**	5(2%)	16(6%)	
**Pathological T classification**			0.225 ^b^
**1**	48(23%)	62(22%)	
**2**	134(63%)	158(57%)	
**3**	24(11%)	50(18%)	
**4**	7(3%)	9(3%)	
**Pathological N classification**			0.410 ^b^
**0**	41(19%)	59(21%)	
**1**	90(42%)	102(37%)	
**2**	59(28%)	76(27%)	
**3**	23(11%)	42(15%)	
**Pathologic stage**			0.274 ^b^
**II**	121(57%)	144(52%)	
**III**	92(43%)	135(48%)	
**Estrogen receptor status**			0.943 ^b^
**Positive**	113(53%)	150(54%)	
**Negative**	83(39%)	109(39%)	
**Uncertain**	17(8%)	20(7%)	
**Progesterone receptor status**			0.835 ^b^
**Positive**	95(45%)	129(46%)	
**Negative**	99(46%)	129(46%)	
**Uncertain**	19(9%)	21(8%)	
**Mean interval (months) between**			
**MRM and RT(range, median)**	6.54(3–10,6)	6.12(1–10,6)	<0.001 ^a^
**MRM and CT(range, median)**	1.25(0–4,1)	0.76(0–4,1)	<0.001 ^a^
**Overall survival (month)**			0.224 ^b^
**<60**	75(35%)	114(41%)	
**≧60**	138(65%)	165(59%)	
**Overall survival (month)**			0.323 ^b^
**<120**	154(72%)	190(68%)	
**≧120**	59(28%)	89(32%)	
**Disease free survival (month)**			0.467 ^b^
**<60**	95(45%)	134(48%)	
**≧60**	118(55%)	145(52%)	
**Disease free survival (month)**			0.609 ^b^
**<120**	158(74%)	200(72%)	
**≧120**	55(26%)	79(28%)	

^a^ By t test

^b^ By Chi-square test

Abbreviations: TRAM: transverse rectus abdominis myocutaneous; MRM: modified radical mastectomy; RT: radiotherapy; CT: chemotherapy

### Local recurrence and distant metastasis

At a mean follow-up time of 7.2 years (86.5 months), the local recurrence rate was 2.4% (6 of 265 patients in stage II, and 6 of 227 in stage III); while the incidence of distant metastases was 20.5% overall (33 of 265 patients in stage II, and 68 of 227 patients in stage III).

As shown in Table 2, local recurrence was seen in 3 patients (1.4%) of the TRAM flap group and 9 patients (3.2%) in the non-flap group (p = 0.136). The higher pathological T classification (p = 0.031) and negative ER status (p<0.001) were associated with local recurrence with statistical significance on univariate analysis. There was no significant difference as to reconstruction, age, laterality, tumor location, pathological cell pattern, pathological nodal classification, stage, PR status, or mean interval to adjuvant CT or RT between the two groups.

**Table 2:** Univariate analyses for risk factors of local recurrence and distant metastasis.

	No local recurrence	Local recurrence	p	No distant metastasis	Distant metastasis	p
**No. of cases**	480(100%)	12(100%)		391(100%)	101(100%)	
**TRAM**			0.136 ^b^			0.736 ^b^
**Yes**	210(44%)	3(25%)		171(44%)	42(42%)	
**No**	220(56%)	9(75%)		220(56%)	59(58%)	
**Mean Age, years (range)**	47.29(27–60)	46.67 (29–56)	0.593 ^a^	47.4(27–60)	46.7(27–60)	0.372 ^a^
**<50**	286(60%)	5(42%)	0.243 ^b^	227(58%)	64(63%)	0.365 ^b^
**≧50**	194(40%)	7(58%)		164(42%)	37(37%)	
**Laterality**			0.256 ^b^			0.435 ^b^
**Left**	245(51%)	4(33%)		194(50%)	55(55%)	
**Right**	235(49%)	8(67%)		197(50%)	46(45%)	
**Location**			>0.999 ^b^			0.262 ^b^
**Lateral**	349(73%)	9(75%)		289(74%)	69(69%)	
**Central/medial**	131(27%)	3(25%)		102(26%)	32(31%)	
**Pathology**			0.871 ^b^			0.704 ^b^
**Infiltrating ductal carcinoma**	420(88%)	10(84%)		342(88%)	88(87%)	
**Infiltrating lobular carcinoma**	33(7%)	1(8%)		25(6%)	9(9%)	
**Medullary carcinoma**	7(1%)	0(0%)		6(2%)	1(1%)	
**Other**	20(4%)	1(8%)		18(4%)	3(3%)	
**Pathological T classification**			0.031 ^b^			0.667 ^b^
**1**	109(23%)	1(8%)		91(24%)	19(19%)	
**2**	286(59%)	6(50%)		232(59%)	60(59%)	
**3**	71(15%)	3(25%)		56(14%)	18(18%)	
**4**	14(3%)	2(17%)		12(3%)	4(4%)	
**Pathological N classification**			0.930 ^b^			<0.001 ^b^
**0**	98(20%)	2(17%)		91(23%)	9(9%)	
**1**	188(39%)	4(33%)		165(42%)	27(27%)	
**2**	131(27%)	4(33%)		91(23%)	44(43%)	
**3**	63(14%)	2(17%)		44(12%)	21(21%)	
**Pathologic stage**			>0.999 ^b^			<0.001 ^b^
**II**	259(54%)	6(50%)		232(59%)	33(33%)	
**III**	221(46%)	6(50%)		159(41%)	68(67%)	
**Estrogen receptor**			0.001 ^b^			0.735 ^b^
**Positive**	262(54%)	1(8%)		212(54%)	51(51%)	
**Negative**	181(38%)	11(92%)		151(39%)	41(41%)	
**Uncertain**	37(8%)	0(0%)		28(7%)	9(8%)	
**Progesterone receptor**			0.115 ^b^			0.886 ^b^
**Positive**	221(46%)	9(75%)		180(46%)	44(45%)	
**Negative**	219(46%)	3(25%)		180(46%)	48(47%)	
**Uncertain**	41(8%)	0(0%)		31(8%)	9(8%)	
**Mean interval between(months)**						
**MRM and RT(range)**	6.31(1–10)	6.09(4–9)	0.580 ^a^	6.35(1–10)	6.12(3–10)	0.115 ^a^
**MRM and CT(range)**	0.98(0–4)	0.90(0–2)	0.708 ^a^	0.95(0–3)	1.09(0–4)	0.055 ^a^

^a^ By independent t test

^b^ By Chi-square test

Abbreviations: TRAM: transverse rectus abdominis myocutaneous; MRM: modified radical mastectomy; RT: radiotherapy; CT: chemotherapy

Also shown in Table 2, distant metastases were seen in 42 patients (19.7%) of the TRAM flap group and 59 patients (21.1%) in the non-flap group (p = 0.736). The pathological N classification (p<0.001), any positive lymph node (p<0.001) and pathological stage III (p<0.001) were negatively associated with distant metastases on univariate analysis. There was no significant difference as to reconstruction, age, laterality, tumor location, pathological cell pattern, pathological tumor classification, ER or PR status, mean interval to adjuvant CT or RT between the two groups.

### Disease free survival and overall survival

Table 3 outlines the multivariate analyses of the DFS of 492 patients. DFS were significantly worse with pathological T classification 3–4 compared to that with T classification 1–2 (p = 0.009), any positive nodal number compared with N0 (p<0.001), and negative ER status (p = 0.001). With or without TRAM flap reconstruction did not affect DFS (HR = 0.817; 95% CI, 0.495 to 1.142).

**Table 3:** Multivariate analyses of 492 breast cancer patients by Cox’s disease free survival and overall survival.

	Disease free survival		Overall survival	
	Hazard ratio (95%CI)	P value	Hazard ratio (95%CI)	P value
**Age(≧50 years vs. <50 years)**	0.733(0.55–1.259)	0.177	0.734(0.471–1.145)	0.173
**Laterality(right vs. left)**	0.853(0.548–1.237)	0.469	0.832(0.543–1.274)	0.398
**Location(lateral vs. medial/central)**	1.157(0.794–1.939)	0.537	1.318(0.826–2.104)	0.247
**Pathological T classification (T3 and T4 vs. T1 and T2)**	1.91(1.166–2.986)	0.009	2.272(1.404–3.676)	0.001
**Pathological N classification (N0 vs. N1-3)**	4.062(1.788–7.309)	<0.001	6.331(2.708–14.799)	<0.001
**Estrogen receptor(positive vs. negative)**	0.491(0.387–0.868)	0.001	0.472(0.309–0.722)	0.001
**TRAM (with TRAM flap reconstruction vs. no reconstruction)**	0.817(0.495–1.142)	0.372	0.791(0.510–1.227)	0.296

Abbreviations: TRAM: transverse rectus abdominis myocutaneous

Table 3 also describes factors associated with OS. OS were significantly worse with pathological T classification 3–4 compared to that with T classification 1–2 (p = 0.001), any positive nodal number compared with N0 (p<0.001), and negative ER status (p = 0.001). With or without TRAM flap reconstruction did not affect OS (HR = 0.791; 95% CI, 0.510 to 1.227).

The 5-year and 10-year DFS were 81% and 76% for the TRAM flap group and 78% and 73% for the non-flap group. The 5-year and 10-year OS were 89% and 73% for the TRAM flap group and 83% and 74% for the non-flap group.

**Fig 1:**
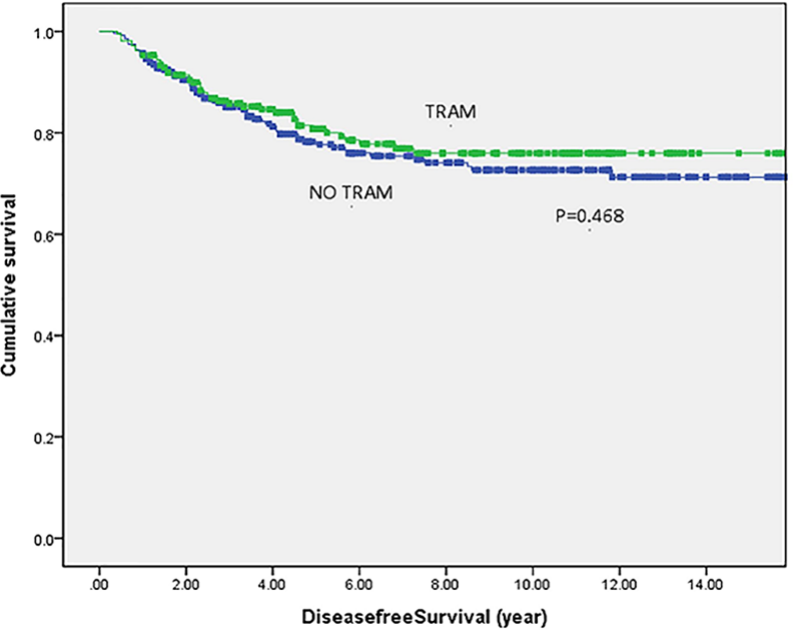
Disease free survival in 492 breast cancer patients. Kaplan-Meier curves demonstrate disease free survival in 492 study patients. Green line denotes TRAM flap group (n = 213) while blue line represents patients with no reconstruction (n = 279). Abbreviation: TRAM: transverse rectus abdominis myocutaneous.

**Fig 2:**
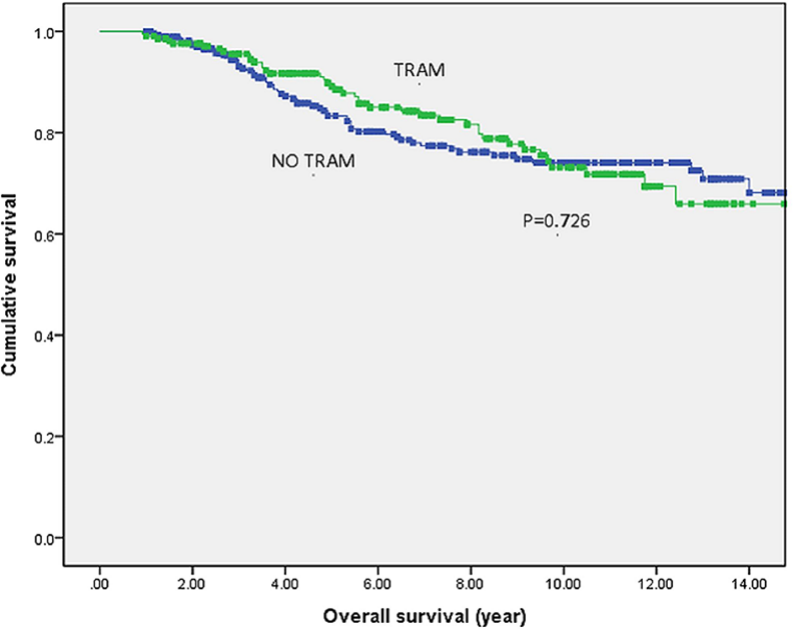
Overall survival in 492 breast cancer patients. Kaplan-Meier curves demonstrate overall survival in 492 study patients. Green line denotes TRAM flap group (n = 213) while blue line represents patients with no reconstruction (n = 279). Abbreviation: TRAM: transverse rectus abdominis myocutaneous.

In Figs 1 and 2, Kaplan-Meier curves demonstrated DFS and OS between both groups. At a mean follow-up time of 86.5 months, there were no significant differences in both DFS rates (p = 0.468) and OS rates (p = 0.726) between both groups.

## Discussion

Postmastectomy reconstruction may be performed either immediately or after a delay, and can use patients’ own tissue, breast implants, or a combination of the two [17]. Patients who had MRM were left with loss of one breast causing detrimental body images and disadvantageous quality of life. Yet abdominoplasty as a part of TRAM flap reconstruction immediately yields positive self-image from the decreased waist circumference. Besides, reconstructed breasts formed by autogenous tissues result in the most natural appearance [8]. Patients with TRAM flap reconstruction often require fewer follow-up office visits than for tissue expansion and require fewer subsequent surgical procedures [8].

Weichman and colleagues reported that patients with a body mass index less than 22 kg/m^2^ have higher satisfaction with their breasts, when compared with those who have prosthetic reconstruction with tissue expander or implant [18]. Wong and colleagues reviewed 62 patients, none of the 38 non-implant patients underwent major corrective surgery within 6 months, compared to three of the 13 (23%) implant patients (p = 0.01) [19]. Jhaveri and colleagues raised the question as to the complications resulting from PMRT in patients after immediate reconstruction. They studied 92 patients and found that the rate of grade 3 or 4 complications (necessitating surgical intervention or removal or replacement of reconstructed breast) was 33.3% for tissue expander or implants versus 0% for autologous tissue (p = 0.001). Acceptable cosmesis was reported in 51% of tissue expander or implants versus 82.6% of autologous tissue reconstruction (p = 0.007) [20].

Berry and colleagues evaluated 1037 patients with immediate reconstruction using either two-stage implant or autologous flaps. In this retrospective study the authors concluded there was no significant difference between the irradiated and non-irradiated autologous tissue reconstructions [21]. Furthermore, Barry and Kell conducted a meta-analysis and identified 1105 patients from 11 appropriately selected studies. They found that when PMRT was delivered after breast reconstruction, autologous reconstruction was associated with less morbidity than implant-based reconstruction [22].

At our institution, immediate reconstruction with autologous tissue using TRAM flap is performed after MRM on the same day for patients who opted to receive reconstruction during preoperative consultation. A retrospective study compared 191 women treated at our institution, all of whom received PMRT [23]. Eighty-two of those patients were reconstructed with an immediate TRAM flap, and the remainder had no reconstruction. At a median follow-up time of 40 months, there was no difference noted in terms of acute skin reactions, between the 2 groups. However, survival was not addressed. This report focused on radiation induced acute dermatitis and provided a rationale for safe use of PMRT after immediate reconstruction. The current study, however, emphasized long-term survival in 492 patients with a mean follow-up period of 86.5 months.

In 2012, Ho and colleagues described 151 patients treated at the Memorial Sloan-Kettering Cancer Center with immediate 2-stage tissue expander and implant reconstruction, CT and PMRT [7]. The 7-year distant DFS was 81%, and the 7-year OS rate was 93%. The 7-year local and regional control was 100%. Yet multivariate analysis was not performed.

Comparing with that of homogeneously treated patients in the current study who received immediate reconstruction followed by CT and then PMRT, the 7-year DFS were 77% and 76% for the TRAM flap group and the non-flap group, respectively. The 7-year OS were 83% and 77% for the TRAM flap group and the non-flap group, respectively.

Alderman et al. assessed 3643 stages I-III breast cancer patients and concluded that immediate postmastectomy breast reconstruction was associated with a statistically significant delay in initiating chemotherapy [24]. The delay induced by the immediate reconstruction remains limited in time and is therefore probably not relevant. In addition, the delay between mastectomy and RT is determined mainly by the duration of the chemotherapy.

In the present study, reconstruction delayed adjuvant therapy significantly (p<0.001). The mean intervals between MRM and CT were 1.25 and 0.76 months in TRAM flap group and non-flap group, respectively (p<0.001). The mean intervals between MRM and RT were 6.54 and 6.12 months in TRAM flap group and non-flap group, respectively (p<0.001) (Table 1). Commonly it was due to waiting for surgical wound healing in the TRAM flap group. However, the longer intervals between MRM and CT or that between MRM and PMRT in the TRAM flap group did not affect the local recurrence (p = 0.708 and p = 0.58, respectively) or distant metastasis (p = 0.055 and p = 0.115, respectively) (Table 2).

Recent literature suggests that breast reconstruction does not negatively influence detection of breast cancer recurrence [14]. In Europe, Veronesi et al. reviewed their series and searched the MEDLINE database for studies of immediate breast reconstruction after mastectomy that were published after the 1990s with a mean follow-up period more than 1 year. They found that DFS and OS were very similar for patients treated by mastectomy only or mastectomy with reconstruction [25]. Kronowitz at al. at the M. D. Anderson Cancer Center pointed out that a tissue expander on the chest wall during radiotherapy does not negatively impact 3-year recurrence free survival [26, 27].

More and more research data shows that breast reconstruction has favorable impact on postmastectomy patient survival. By utilizing data from the US National Cancer Institute’s Surveillance, Epidemiology, and End Results (SEER) registries, Bezuhly et al. found that improved breast cancer-specific survival was observed among all immediate breast reconstruction patients compared with patients who underwent mastectomy alone (HR = 0.74; 95% CI, 0.68 to 0.80). Autologous reconstruction was associated with improved cancer-specific survival among patients below the age of 50 (HR = 0.58; 95% CI, 0.42 to 0.80) and between ages 50 to 69 (HR = 0.61; 95% CI, 0.43 to 0.85) [16]. In the current study, the mean age at diagnosis was 44.8 (range 27–60) years in the TRAM flap group and 49.1 (range 27–60) years in the non-flap group. (Table 1)

Agarwal et al performed multivariate statistics from the SEER database which revealed that patients treated with mastectomy and reconstruction had a significantly lower hazard ratio of death (HR = 0.62, p < 0.001) compared with patients treated with mastectomy only, when controlling for demographic and oncologic covariates [15]. Dr. Jayant Agarwal and colleagues further investigated a cohort of 52,249 patients from the SEER database and noted that patients treated with mastectomy and reconstruction had a significantly lower hazard of death (HR = 0.73, p < 0.0001) compared with patients treated with mastectomy only. Radiotherapy was not significantly associated with hazard of death (HR = 1.03, p = 0.3494) [14].

In the present study, the authors compared the long-term clinical outcomes in a cohort of 492 patients who underwent PMRT with and without prior immediate TRAM flap reconstruction at a single cancer center in Asia. The cosmetic outcome and complication rates were not the study endpoints. Recurrence and survival were the focus. The authors found no significant DFS or OS difference, and no significantly different local recurrence or distant metastases rates between patients with and without TRAM flap reconstruction.

The pitfall of the current study includes inherent biases in a retrospective study. Because of the patients’ preference, such a study cannot be randomized. This is the major limitation. Besides, the percentage of patients achieving good, fair and poor cosmesis was not assessed. We mainly focused on the result of recurrence and survival. Information such as co-morbidity (diabetes; vascular problems) and smoking habits were not complete in our own hospital records. The lack of data regarding HER2/neu (human epidermal growth factor receptor 2) amplification was another drawback. The detail of oncogene could be important in assess risk factors in local recurrence, distant metastases and even survival.

## Conclusion

Immediate TARM flap reconstruction prior to PMRT does not compromise breast cancer patients’ survival. These long-term outcomes provide implications for both the physicians and patients who come to a decision concerning immediate TRAM flap reconstruction followed by CT and PMRT.
